# Management of Immature Permanent Mandibular First Molar Using NeoPutty: A Case Report

**DOI:** 10.1002/ccr3.71302

**Published:** 2025-10-20

**Authors:** Maryam Khorasanchi, Maryam Gharechahi, Zahra Azizi

**Affiliations:** ^1^ Department of Endodontics, Faculty of Dentistry Mashhad University of Medical Sciences Mashhad Iran; ^2^ Department of Restorative Dentistry, Faculty of Dentistry Mashhad University of Medical Sciences Mashhad Iran

**Keywords:** apexification, calcium silicate, fistula, odontogenic infections, periapical diseases

## Abstract

Facial cutaneous sinus tracts of odontogenic origin are often misdiagnosed and improperly managed. An 8‐year‐old girl presented with a draining submental sinus tract caused by a periapical infection of her immature permanent mandibular first molar. Given the open apices and prolonged infection, apexification was planned. NeoPutty, a recently introduced premixed bioactive bioceramic cement composed primarily of tricalcium and dicalcium silicate, was selected for apical plug placement. NeoPutty has demonstrated favorable biocompatibility and antibacterial properties and has been successfully applied in primary molar pulpotomies and indirect pulp therapy. In this case, both the periapical lesion and the extraoral sinus tract healed successfully within 6 months, outcomes attributable to the material's biological and antibacterial characteristics.

## 1 Introduction

Managing immature permanent teeth with necrotic pulp presents significant clinical challenges. The absence of an apical stop increases the risk of inadequate sealing, incomplete instrumentation, and eventual treatment failure. Apexification has long been the standard approach for such cases, either by inducing a calcified barrier at the open apex or by encouraging further apical development in incompletely formed roots [1].

The apical plug technique is widely used in these scenarios, as it provides a reliable apical barrier while reducing treatment duration compared with traditional long‐term calcium hydroxide therapy. Among available materials, mineral trioxide aggregate (MTA) has become the gold standard. Composed of tricalcium silicate, tricalcium aluminate, tricalcium oxide, and silica, MTA hydrates to form a colloidal gel that sets into a rigid structure [2]. It performs well in moist environments, has a high pH, and promotes cementitious tissue formation, thereby supporting the development of a biological seal [2]. However, MTA's handling is difficult; its gritty texture complicates placement and compaction, and its reliance on precise powder–liquid ratios makes it sensitive to mixing errors. These shortcomings can compromise consistency, setting, and sealing ability, prompting the search for alternatives [3].

NeoMTA Plus (Avalon Biomed Inc., Bradenton, USA) was developed to overcome some of these limitations. It uses tantalum oxide instead of bismuth oxide as a radiopacifier, eliminating the discoloration commonly associated with MTA [4, 5]. More recently, NeoPutty, a premixed formulation, was introduced. It contains tricalcium silicate, dicalcium silicate, tricalcium aluminate, calcium aluminate, calcium sulfate, and tantalum oxide, offering greater handling convenience and reducing the risk of mixing‐related errors.

Preclinical and clinical studies support the potential of NeoPutty. In vitro, it has demonstrated cytocompatibility with human dental pulp cells comparable to NeoMTA Plus and MTA Angelus, though it releases less calcium than MTA, which may influence bioactivity [6]. A randomized clinical trial reported a 97.1% clinical success rate and a 92.8% radiographic success rate for NeoPutty in primary molar pulpotomies at 12 months, similar to NeoMTA [7]. For indirect pulp therapy in deciduous teeth, it achieved a 91.67% success rate after 6 months, outperforming calcium hydroxide and showing results comparable to Biodentine [8]. Additionally, a case report described successful revascularization using NeoPutty in immature maxillary lateral incisors following trauma, with apical closure observed [9]. Despite these promising findings, the use of NeoPutty specifically as an apical plug material for apexification has not yet been reported in the literature. This case report describes the successful management of a necrotic immature permanent mandibular first molar in an 8‐year‐old patient, highlighting NeoPutty's potential as a novel alternative for apexification procedures.

## 2 Case History/Examination

**FIGURE 1:**
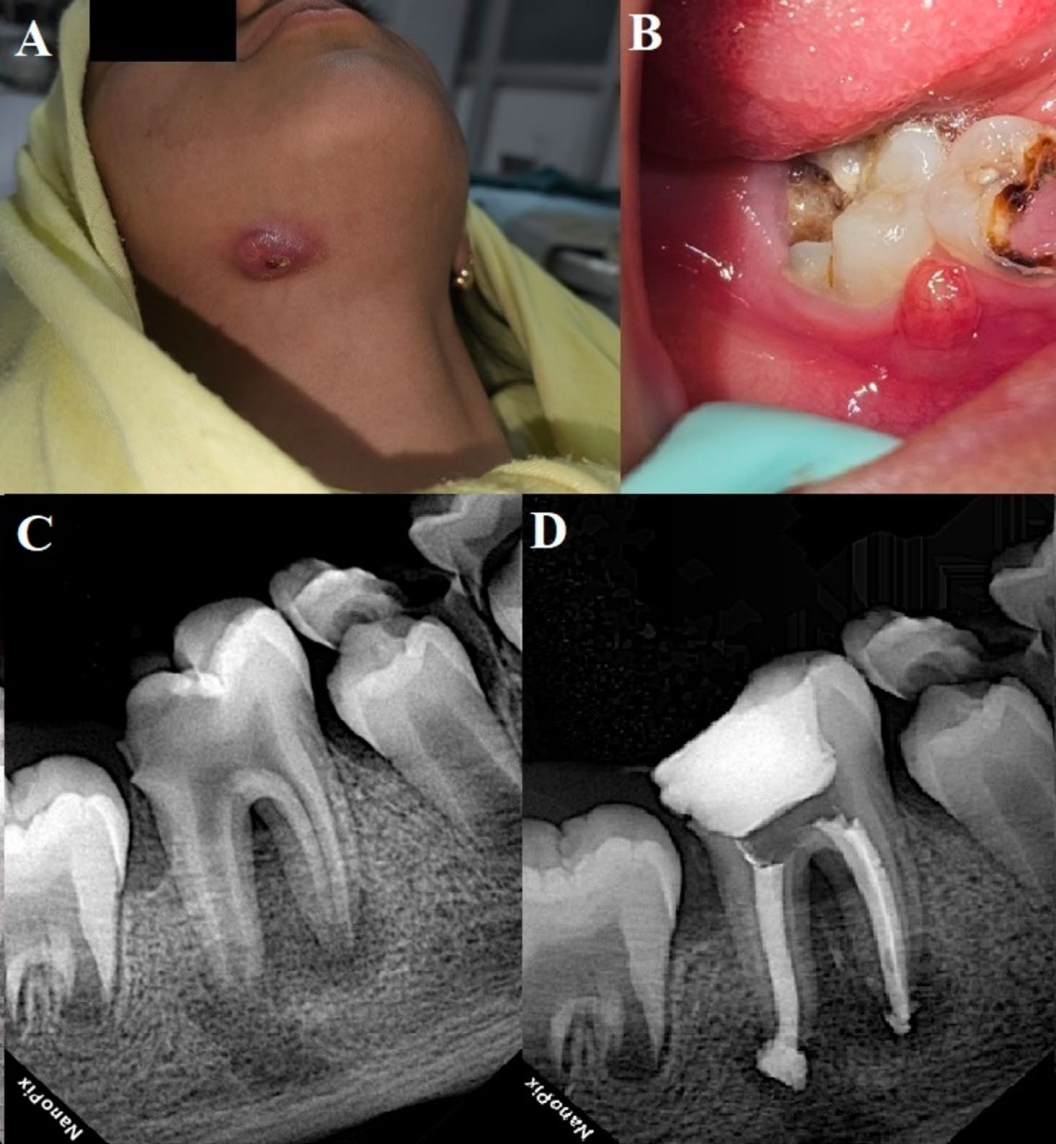
(A) Extraoral view of the patient showing a draining chronic cutaneous sinus tract. (B) Intraoral examination revealed a fistula adjacent to the severely carious mandibular first molar. (C) The initial periapical radiograph demonstrated a large periapical radiolucency associated with incomplete root formation. (D) An immediate postoperative radiograph showed the outcome following apexification with NeoPutty and obturation using thermoplastic gutta‐percha.

An 8‐year‐old girl presented to the Endodontics Department at Mashhad University of Medical Sciences with a chief complaint of a draining submental cutaneous sinus tract (Figure 1A). Her medical history was unremarkable. According to her parents, the initial consultation with a physician included an ultrasound, which revealed scattered reactive lymph nodes in the right submental and submandibular regions; however, the findings were inconclusive. Subsequent medical evaluations raised concern for malignancy, and a complete blood test was ordered. Eventually, one physician referred the patient for a dental examination.

Intraoral examination revealed carious exposure of the right permanent mandibular first molar, accompanied by an intraoral sinus tract (Figure 1B). The tooth was tender to palpation and percussion. Both the cold test and the electric pulp test were negative. A periapical radiograph showed significant caries, an associated periapical lesion, and incompletely developed root apices (Figure 1C). Based on these findings, the tooth was diagnosed with pulpal necrosis and chronic apical abscess. Given the immature apices, apexification with apical plug placement was planned. Regenerative endodontic treatment (RET) was not considered, as persistent infection is known to reduce its success rate [10]. After discussing the treatment plan and prognosis with the patient's guardian, written informed consent was obtained.

## 3 Methods (Differential Diagnosis, Investigations and Treatment)

For the apexification procedure, local anesthesia was administered via inferior alveolar block using 2% lidocaine with 1:80,000 epinephrine (Darou Pakhsh Pharmaceutical Manufacturing Co., Tehran, Iran). The tooth was isolated with a rubber dam, caries were excavated with a round bur, and an access cavity was prepared using a high‐speed fissure bur. Working length was determined with an apex locator (Propex IQ, Dentsply Maillefer, Ballaigues, Switzerland) and confirmed radiographically. The canals were minimally prepared using the ProTaper Universal system (Dentsply Maillefer, Ballaigues, Switzerland) with F3 files in a brushing motion to remove necrotic pulp and biofilm from the canal walls [11].

Irrigation was performed with 2.5% sodium hypochlorite (NaOCl) using side‐vented needles placed 2–3 mm short of working length, with activation by an EndoActivator (Dentsply, Tulsa, OK, USA). The canals were finally rinsed with saline and dried with paper points. The apical foramina sizes corresponded to gutta‐percha cones of sizes 65 (mesiobuccal), 55 (mesiolingual), and 80 (distal).

After confirming the absence of intracanal exudate, premixed NeoPutty (Avalon Biomed Inc., Bradenton, USA) was used to create 3–5 mm apical plugs. Material was dispensed onto a slab and delivered into the canals with the MAP One system (PD Dental, Switzerland). A moist paper point and temporary restoration were placed, and the patient was discharged to allow the material to set.

At the 24‐h recall, the temporary filling was removed, the canals were irrigated, and the NeoPutty was checked with an endodontic file to confirm complete setting. The canal walls were coated with AH 26 sealer (Dentsply, Tulsa, OK, USA), and obturation was completed using the warm vertical compaction technique with a FastFill obturator (Eighteeth, China). A temporary restoration was placed (Figure 1D), and the patient was referred to the Pediatric Dentistry Department for final restoration.

The tooth was permanently restored with amalgam and a preformed stainless steel crown (SSC; 3M, Minnesota, USA). Occlusion and periodontal status were evaluated, and oral hygiene instructions were provided. The patient was scheduled for regular follow‐up.

## 4 Conclusion and Results (Outcome and Follow‐Up)

**FIGURE 2:**
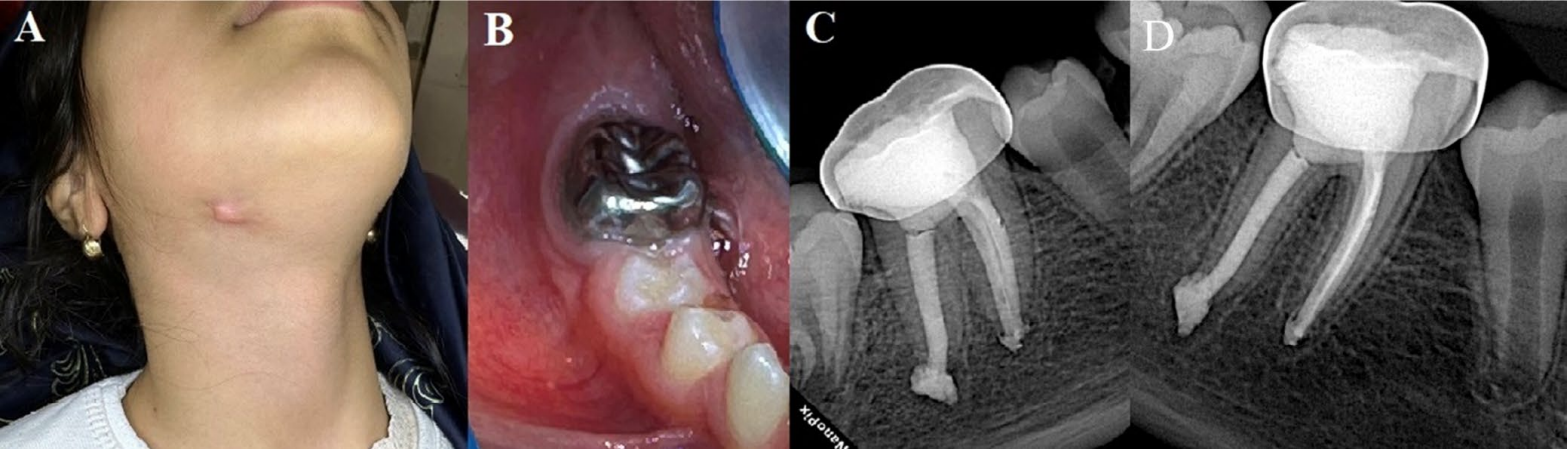
(A) Clinical follow‐up revealed healing of the extraoral sinus tract with residual scar tissue and (B) a functional tooth with healthy gingival tissues. (C) A 6‐month and (D) 12‐month follow‐up radiograph demonstrated substantial reduction of the periapical radiolucency and reestablishment of a normal periodontal ligament space around the roots.

At both the 6‐ and 12‐month recalls, the patient remained asymptomatic, and clinical examination revealed no signs of endodontic complications. Periapical radiographs taken at these follow‐ups showed progressive reduction of the lesion, evidence of bone fill, and reestablishment of a normal lamina dura around both roots (Figure 2).

## 5 Discussion

Cutaneous sinus tracts of dental origin are often misdiagnosed when patients initially present to physicians [12, 13]. Such errors can result in unnecessary biopsies, prolonged or inappropriate antibiotic use, and even radiation therapy [14]. Dental infection should always be considered in cases of persistent drainage in the head and neck region [15, 16]. Typically, pulpal necrosis initiates periapical inflammation and osteoclastic bone resorption. The resulting exudate may remain intraosseous or extend beyond cortical bone, producing cellulitis or abscesses [14]. Mandibular teeth are most frequently implicated, with drainage appearing through the chin or mandibular angle [14]. In this case, the patient endured 6 months of drainage, unnecessary medical investigations, and even suspicion of malignancy before the dental cause was identified.

Successful management requires elimination of the primary infection through either root canal treatment or extraction, depending on tooth prognosis. Once the source is removed, sinus tracts generally resolve within 5–15 days without antibiotics, leaving only minimal scarring [17]. The observations, consistent with other reports [14, 18], reinforce the importance of accurate diagnosis and timely endodontic intervention.

For immature permanent teeth with necrotic pulps, apexification remains a well‐established treatment, particularly when RET is contraindicated by persistent infection or damaged apical tissues [10, 19]. In this case, a 12‐month follow‐up revealed radiographic bone fill, reestablishment of lamina dura, and absence of symptoms, confirming periapical healing. Although signs of improvement may emerge within 3–6 months, a 12‐month interval is generally considered the minimum reliable period to confirm healing [20]. Continued monitoring is advisable to ensure long‐term stability.

NeoPutty was selected as the apical plug material in this case. Introduced in 2020, it was designed to overcome limitations of MTA, particularly discoloration due to bismuth oxide. NeoPutty uses tantalite as its radiopacifier, eliminating this risk [2]. It is indicated for a broad range of endodontic applications, including pulp capping, pulpotomy, apexification, perforation repair, and root‐end filling. Its performance is supported by in vitro evidence of high biocompatibility with pulp and periodontal cells [6, 21, 22], and its calcium aluminate content has demonstrated favorable tissue responses in vivo [23] and osteogenic potential in vitro [24].

Compared with other modern bioceramics, NeoPutty offered practical advantages that guided its selection. Biodentine has excellent clinical outcomes but requires powder‐liquid mixing in a triturator, making it more technique‐sensitive; improper mixing or air inclusion can compromise adaptation, especially in wide or irregular apices [25]. iRoot FS is premixed and biologically favorable [26], but its rapid setting kinetics reduce working time, limiting the ability to adjust or compact the plug in anatomically challenging situations [27]. NeoPutty, by contrast, is premixed, firm, non‐tacky, and resistant to wash‐out, providing predictable handling and reducing the risk of displacement during placement in open apices [7]. MTA, while historically successful, presents handling difficulties. Its mud‐like consistency complicates placement, and when improperly mixed, its sandy texture and lack of cohesiveness increase wash‐out risk [28, 29]. NeoPutty avoids these drawbacks, offering simplified handling without the risk of mixing errors.

In addition to handling advantages, NeoPutty's alkaline pH contributes to antimicrobial effects [30]. Unlike MTA, which shows limited activity against *Enterococcus faecalis* [30], NeoPutty demonstrates activity against *Pseudomonas aeruginosa* and acceptable efficacy against other oral pathogens, including *E. faecalis* , *Staphylococcus aureus* , and *Escherichia coli* [30]. Cruz Hondares et al. [31] further confirmed its antimicrobial and mineralization‐supporting capacity alongside established materials such as ProRoot MTA, Biodentine, and NeoMTA 2.

Clinically, NeoPutty can be immediately restored or crowned after placement since it hardens securely beneath restorations, resists wash‐out, and maintains dimensional stability even in acidic or alkaline environments [32]. Taken together, these properties make NeoPutty a practical and reliable material for apexification in immature permanent teeth. Its premixed formulation, favorable handling, bioactivity, and antibacterial potential explain the successful outcomes achieved in this case.

## 6 Conclusion

Facial cutaneous sinus tracts originating from odontogenic issues are frequently misdiagnosed and treated incorrectly. This case report describes the successful management of an immature mandibular first molar with a cutaneous sinus tract using NeoPutty for apexification. Treatment resulted in the resolution of the sinus tract and radiographic periapical healing within 12 months, achieved without antibiotics or surgical intervention. The novelty of this case lies in documenting the first clinical use of NeoPutty as an apical plug material. Its premixed formulation, biocompatibility, antibacterial properties, and ease of handling offer distinct advantages over traditional MTA, which is more technique‐sensitive. These characteristics suggest NeoPutty may be a practical and predictable alternative in cases where regenerative therapy is contraindicated and secure apical sealing is essential. While these findings are promising, the limitation of a single case must be acknowledged. Larger clinical studies with extended observation are necessary to validate NeoPutty's long‐term effectiveness in apexification procedures.

## Author Contributions

**Maryam Khorasanchi:** investigation, methodology. **Maryam Gharechahi:** conceptualization, supervision, writing – review and editing. **Zahra Azizi:** investigation, methodology, writing – original draft.

## Ethics Statement

The authors have nothing to report.

## Consent

A written consent form was obtained from the patient's parents for radiographs and other clinical information to be reported in the journal. The patient's parents understood that the patient's name and initial would not be published.

## Conflicts of Interest

The authors declare no conflicts of interest.

## Data Availability

Data sharing not applicable to this article as no datasets were generated or analysed during the current study.
